# MicroRNAs: Potential mediators between particulate matter 2.5 and Th17/Treg immune disorder in primary membranous nephropathy

**DOI:** 10.3389/fphar.2022.968256

**Published:** 2022-09-21

**Authors:** Xiaoshan Zhou, Haoran Dai, Hanxue Jiang, Hongliang Rui, Wenbin Liu, Zhaocheng Dong, Na Zhang, Qihan Zhao, Zhendong Feng, Yuehong Hu, Fanyu Hou, Yang Zheng, Baoli Liu

**Affiliations:** ^1^ Beijing University of Chinese Medicine, Beijing, China; ^2^ Beijing Hospital of Traditional Chinese Medicine, Capital Medical University, Beijing, China; ^3^ Shunyi Branch, Beijing Hospital of Traditional Chinese Medicine, Beijing, China; ^4^ Beijing Institute of Chinese Medicine, Beijing, China; ^5^ School of Life Sciences, Beijing University of Chinese Medicine, Beijing, China; ^6^ School of Traditional Chinese Medicine, Capital Medical University, Beijing, China; ^7^ Pinggu Hospital, Beijing Hospital of Traditional Chinese Medicine, Beijing, China; ^8^ School of Traditional Chinese Medicine, Changchun University of Chinese Medicine, Changchun, China

**Keywords:** PM2.5, PLA2R1, microRNA, Th17/Treg, primary membranous nephropathy (PMN)

## Abstract

Primary membranous nephropathy (PMN), is an autoimmune glomerular disease and the main reason of nephrotic syndrome in adults. Studies have confirmed that the incidence of PMN increases yearly and is related to fine air pollutants particulate matter 2.5 (PM2.5) exposure. These imply that PM2.5 may be associated with exposure to PMN-specific autoantigens, such as the M-type receptor for secretory phospholipase A2 (PLA2R1). Emerging evidence indicates that Th17/Treg turns to imbalance under PM2.5 exposure, but the molecular mechanism of this process in PMN has not been elucidated. As an important indicator of immune activity in multiple diseases, Th17/Treg immune balance is sensitive to antigens and cellular microenvironment changes. These immune pathways play an essential role in the disease progression of PMN. Also, microRNAs (miRNAs) are susceptible to external environmental stimulation and play link role between the environment and immunity. The contribution of PM2.5 to PMN may induce Th17/Treg imbalance through miRNAs and then produce epigenetic affection. We summarize the pathways by which PM2.5 interferes with Th17/Treg immune balance and attempt to explore the intermediary roles of miRNAs, with a particular focus on the changes in PMN. Meanwhile, the mechanism of PM2.5 promoting PLA2R1 exposure is discussed. This review aims to clarify the potential mechanism of PM2.5 on the pathogenesis and progression of PMN and provide new insights for the prevention and treatment of the disease.

## 1 Introduction

Membranous nephropathy (MN) is a pathological pattern of primary glomerular disease and has developed into the main pathological type of adult nephrotic syndrome. Primary membranous nephropathy (PMN) is a part of membranous nephropathy with unknown etiology, accounting for about 75% (Lai et al., 2015). The occurrence of PMN results from complex interactions of environment, genetics, and immunity as a classic model of autoimmune glomerular disease. However, the specific mechanism of air pollution components and risk loci inducing PMN pathological injury pattern formation has not been confirmed. About 25% of the remaining patients are secondary MN, associated with various diseases such as malignancy, systemic lupus erythematosus, drug reactions, and infections (Akiyama et al., 2019). Unlike other kidney diseases, the incidence of MN has been substantially increasing in recent 10 years. MN has passed IgA nephropathy to become the leading cause of adult nephrotic syndrome (Hu et al., 2020). Spontaneous remission of proteinuria occurs in 30%–40% of patients, while slow progression to end-stage renal disease (ESRD) occurs in the remaining patients after 5–15 years (Lai et al., 2015). Early diagnosis and reasonable intervention of PMN are essential.

The clinical manifestations of PMN are mostly nephrotic syndrome or asymptomatic proteinuria. And its pathology is characterized by diffuse thickening of the basement membrane. Its immunofluorescence is a diffuse granular deposition of IgG and complements C3 on glomerular capillary walls, with IgG4 being the predominant IgG subtype (Fogo et al., 2015; Ahmad and Appel, 2020). At present, the renal biopsy result is used as the gold standard for diagnosing MN. Pathogenic podocyte autoantigens can be found in the glomerular immune deposits as the incentives to stimulate the formation of *in situ* immune complexes (Couser et al., 1978). As pro-inflammatory factors of autoimmune response, helper T (Th) cells promote B cell differentiation and antibody production upstream. In contrast, regulatory T (Treg) cells inhibit this effect and maintain immune tolerance. The formation of initial damage may also further promote the expression of autoantigens and trigger new immune activities (Ronco and Debiec, 2012), yet that hypothesis has not been confirmed. MN recurrence after renal transplantation may be related to circulating autoantibodies reaching the renal binding donor autoantigens (Stahl et al., 2010). These antigens are essential for disease diagnosis, disease progression, and prognosis. Among them, PLA2R1 accounts for about 70% and acts as a characteristic and diagnostic autoantigen of PMN. Some evidences show that PLA2R1 can reflect disease immune activity and is more sensitive and quicker than proteinuria remission, which also indicates that PMN immune remission occurs before clinical remission (Beck et al., 2011; Du et al., 2014). There are good reasons to speculate that the rising incidence of PMN is inextricably linked to increased exposure to self-antigens. From an epidemiological point of view, there are regional differences in disease incidence. It can also be found in clinical practice that some PMN patients still relapse after the regular application of immunosuppressive therapy. Our team proposed that fine air pollutants PM2.5 act as an unstable factor to stimulate continuous autoantigen exposure and lead to autoimmune disorders (Liu et al., 2019). Thus, autoantibodies against ectopic antigens bind to situ antigens in podocytes. This review will also introduce several hypotheses about how PM2.5 causes antigen exposure. Th17/Treg imbalance has been confirmed of great significance in PMN. Correlation studies have shown that PM2.5 exposure relocates the immune response to the Th17 immune pathway (Cremoni et al., 2020). The effect of PM2.5 on Th17/Treg immune balance has been confirmed to have multiple tracks, such as polycyclic aromatic hydrocarbons (PAH), NF-κB, Notch, et al. These intermediate links interact with microRNAs. In multi-system such as respiratory and cardiovascular, the response of microRNA to external pollution stimuli significantly affects Th17/Treg balance. PM2.5 regulating Th17/Treg from upstream *via* microRNA is also considered to be important in PMN. In PMN, antigen exposure by PM2.5 ultimately targets the glomerulus. This feature is different from other types of diseases. Alterations of miRNAs in this process may contribute to this property.

## 2 Primary membranous nephropathy

### 2.1 The exposure of primary membranous nephropathy autoantigens

Heymann et al. (1959) proposed an active Heymann nephritis model. By intraperitoneal injection of the supernatant of rat kidney extract, the animal model exhibits a more pronounced MN-like clinical response (Heymann et al., 1959). Couser et al. (1978) confirmed the existence of antigens on human podocytes as the target of circulating autoantibodies. It is speculated that the pathological model of PMN also has the process of antigen-antibody binding. Beck et al. (2009) found that PLA2R1 is present in normal podocytes and immune deposits of PMN patients. PLA2R1 triggers autoimmune responses under its special intramolecular disulfide bond configuration and colocalizes with IgG (Beck et al., 2009). Anti-PLA2R1 antibodies are found in 70%–80% of patients from different ethnic groups (Du et al., 2014). Autoantigens such as THSD7A (Tomas et al., 2014), Nell-1 (Sethi et al., 2020), and Exostosin1or Exostosin2 (Sethi et al., 2019) were subsequently identified. The pathogenesis of PMN is associated with antibodies binding to podocyte autoantigens. However, PMN-specific autoantigens are not only expressed in podocytes. As early as 2017, a study on PLA2R1 and asthma found that PLA2R1 is expressed in endobronchial tissue and epithelial brushings (Nolin et al., 2016); In 2018, a clinical study in Hamburg, Germany, confirmed that two antigens, PLA2R1 and THSD7A, are stably expressed in bronchioles and podocytes (von Haxthausen et al., 2018). And PLA2R1 expression is not limited to PMN patients. It is also less expressed in normal podocytes (Beck et al., 2009).

Academician Hou Fanfan’s team discovered the relationship between PM2.5 concentration and the increased incidence of PMN in 2016 (Xu et al., 2016). Based on a large number of studies in China, Zhang et al. (2018a) concluded that the growth trend of the disease is closely related to gene-environment interactions. According to our hypothesis of increased PLA2R1 autoantigen exposure under environmental pollution (Liu et al., 2019), extrarenal autoantigens may trigger circulating antibody production.

**FIGURE 1:**
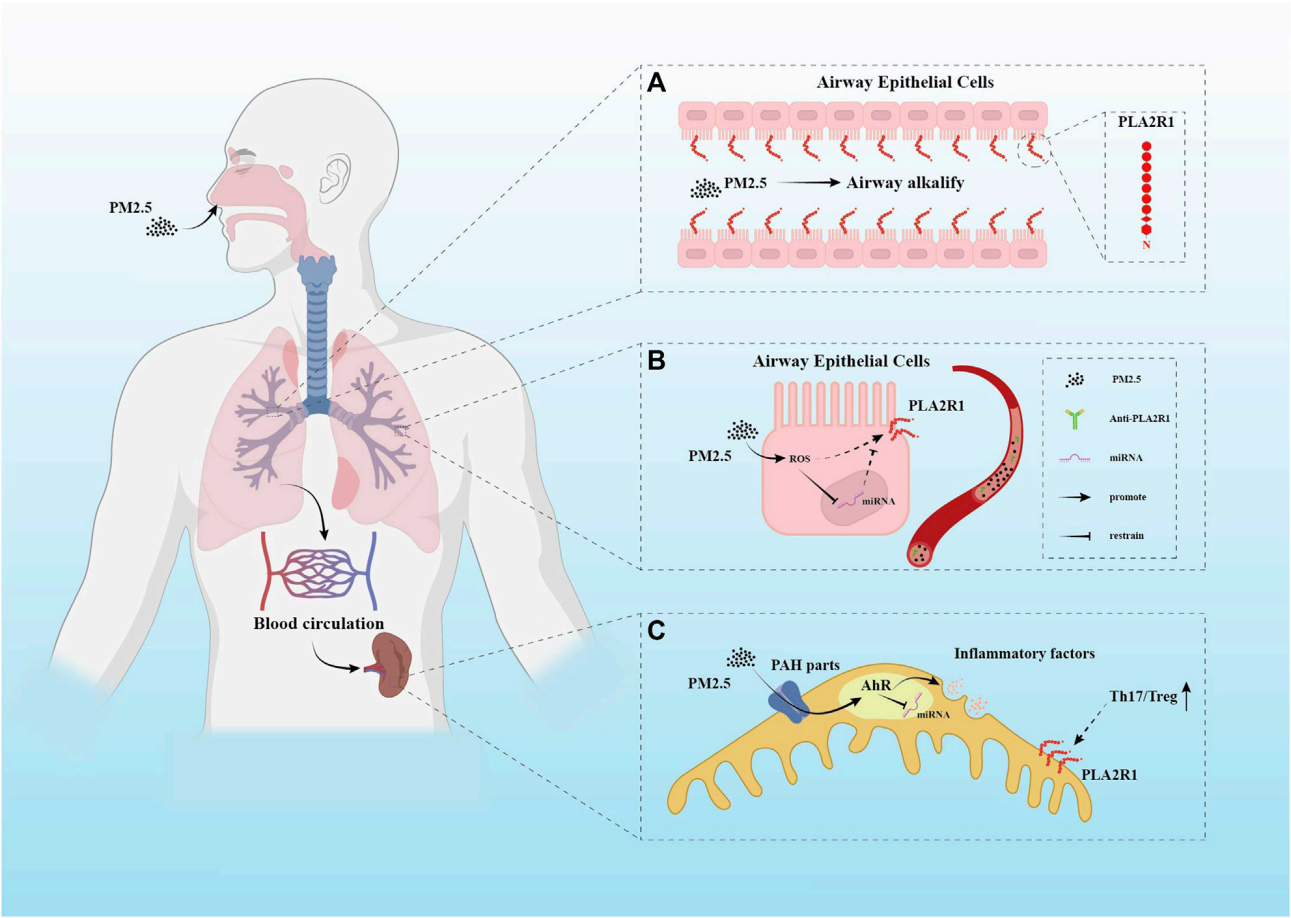
Several different hypothetical mechanisms of PM2.5 pathogenicity. **(A)** PLA2R1 has been demonstrated to be expressed in human respiratory epithelial cells. PM2.5 is mainly composed of polycyclic aromatic hydrocarbons (PAHs) and metals. After entering the respiratory tract, the alkalinization effect of PM2.5 on the airway may enhance the expression of PLA2R1. This conjecture has not been supported by direct evidence. **(B)** The oxidative stress response caused by PM2.5 in the respiratory system may cause changes in miRNAs. The promotion of ROS and the inhibition of miRNAs by PM2.5 may increase the expression of PLA2R1. **(C)** PAH components in PM2.5 have been shown to play a role in the imbalance of Th17/Treg ratio. This component uses AhR as a channel. It is not clear how AhR affects the enhancement of Th17 immune pathway, but miRNAs may be one of them.

MiRNAs are sensitive to external stimuli. A study found that downregulation of miR-130a-5p in MN kidney biopsy specimens promoted podocyte apoptosis. The same phenomenon occurs in a model of AngII-induced podocyte injury. Downregulated miR-130a-5p targeting increases the expression of PLA2R1 (Liu et al., 2018). miR-130a-5p is sensitive to oxidative stress induced by external stimuli. And miR-130a-5p is upregulated in an H_2_O_2_-induced oxidative stress environment (Ayaz and Dinç, 2018). However, there is no direct evidence to prove the variation of miR-130a-5p under PM pollutants. It is known that under the contamination of PM, bronchial epithelial cells can cause significant oxidative stress, and the oxidative stress effect of PM2.5 seems to change with the seasons (Longhin et al., 2016). It is speculated that airborne fine particle pollutants represented by PM2.5 can alter microRNA expression, thereby promoting the exposure of PMN autoantigens. Combined with previous studies, this process may be achieved through oxidative stress (Figure1B), but it has not been confirmed. The entry of PM2.5 into the human body can cause increased levels of oxidative stress. Epigenetic changes often accompany this process. Eleonora Longhin et al. (2016) confirmed the link between oxidative stress and related miRNAs under the molecular network activated by PM2.5; Jin Li et al. (2018a) found that PM2.5 intervention in human alveolar epithelial cells led to increased reactive oxygen species (ROS) generation with downregulation of miR-486. And upregulation of miR-486 can inhibit oxidative stress response; In 2016, Rodosthenis S. Rodosthenous et al. (2016) studied the effect of PM2.5 on the abnormal miRNA expression of extracellular vesicles, which means circulating miRNAs. They demonstrated that PM2.5 exposure-related miRNAs in the circulation are associated with oxidative stress, inflammation, and arteriosclerosis pathways in cardiovascular disease populations. This also led us to think that PM2.5 could not only bind to the important related protein Dicer during miRNAs production to hinder pre-miRNA maturation (Izzotti and Pulliero, 2014), but also affect the function of circulating miRNAs at the post-mature level and cause systemic responses. However, PLA2R1 is not static, and epitope spread occurs as the disease progresses. Moreover, this epitope expansion is associated with renal prognosis stratification in PMN patients (Seitz-Polski et al., 2016). After discovering the relationship between disease activity and epitope spread, no studies have identified factors or pathways associated with changes in the PLA2R1 epitope in PMN.

### 2.2 Major immune pathways of primary membranous nephropathy

The characterization of PMN as an autoimmune disease is the activation of Th17, Th2, and Treg, et al. Research about PM2.5 currently focuses on its contribution to the incidence of PMN and induction of Th17/Treg balance dysregulation. However, the specific immune process of PM2.5 particles entering the body has not been elucidated. PM2.5 particles enter the lung from the respiratory tract and can reach the bottom of the alveoli due to their tiny diameter. Parts of it are taken up and processed by innate immune cells. Others can enter the blood circulation and act on the whole body (Krauskopf et al., 2018). Inflammation and oxidative stress caused by PM2.5 take place through multiple pathways. Although dendritic cells (DCs) are potent antigen-presenting cells (APCs), they can monitor the environment, uptake antigens, and initiate new CD4^+^ T cells in lymph nodes (Hilligan and Ronchese, 2020). However, its role in the entry of PM2.5 into the body is unclear. PM2.5 particle stimulation can induce increased expression of DEC205, a surface receptor present in APCs such as DCs and macrophages, which can influence the maturation of APCs (Honda et al., 2021). However, Zheng et al. (2020) showed that although PM2.5 increased the proportion of mature DCs, it did not enhance the response of lymphocytes, yet it could stimulate the Th17 phenotype. We speculate that PM2.5 particles may not only be taken up and presented by DCs after entering the body. Other APC types may be involved in PM2.5-activated CD4^+^ T cells differentiating into Th17. Changes in macrophages seem to play an important role in studies of immune cell responses to PM2.5. The survey of Soi Jeong et al. (2019) flow cytometry showed that pulmonary macrophages significantly increased after PM2.5 treatment. Taking the liver as an example, it has been proved that PM2.5 particles can cause secondary organ inflammation through blood exposure after entering the human body from the respiratory tract. Fu et al. (2020) demonstrated that PM2.5 could activate macrophages in a dose-dependent manner and cause respiratory system inflammation through TLR4/NF-κB/COX-2 signaling pathway. Macrophages are critical to PM2.5 disrupting immune homeostasis. After being stimulated by foreign pollutants, macrophages play a role in phagocytosis and release inflammatory cytokines, such as NF-κB, and increase the level of oxidative stress (Bekki et al., 2016). Several studies have confirmed that PM2.5 can upregulate the expression of NF-κB, IFN-γ, et al. (Table 1). These phenomena also further confirmed the role of macrophages in PM2.5 particle uptake and Th17/Treg immune imbalance. PM2.5 mediates IFN-γ production. Th17-related immune pathways are enhanced after IFN-γ-induced macrophage activation. In human autoimmune diseases, IFN-γ can target APC to promote the expression of IL-17 (Kryczek et al., 2008). The effects of PM2.5 particles on the polarization of the Th17 immune pathway are not limited to this pathway. Sun et al. (2020) demonstrated that the PAH components in PM2.5 can directly act on CD4^+^ naive T cells from the spleen through aromatic hydrocarbon receptors (AhR) to affect Th17 and Treg immune pathways. And the most apparent different expression of this process is the environmental information processing related genes. PM2.5 particles, especially their adsorbed PAH components, can directly cause changes in the proportion of immune cells in the spleen, increase T cells and decrease B cells (Honda et al., 2017).

**TABLE 1:** The effects of PM2.5 treatment on microRNA expression in different models and the changes of corresponding immune inflammatory factors.

Model	microRNA change	Changes in inflammatory factors	References
BALB/c mice	miR-224↓	Treg↓/Th17↑,TLR2/TLR4/MYD88↑	Ping Li et al. (2020)
BALB/c mice	miR-146↑	IL-6, IFN-γ, TNF-α↑	Hou et al. (2021)
ApoE^−/−^ mice	miR-326-3p↑	NF-κB↑	Gao et al. (2022)
Beas-2B cells	miR-331↓	NF-κB, IL-6, IL-8↑	Song et al. (2017)
HAEC	miR-939-5p↓	HIF-1α↑	Liang et al. (2021)
BALB/c mice	10 miRs	Th1↑/Th2↓, IL-4, IFN-γ↑	Hou et al. (2018)
COPD mice	miR-149-5p↓	NF-κB↑	Qiuyue Li et al. (2021)
A549 cells	miR-582-3p↑	Wnt/β-catenin↑	Yang et al. (2022)
16HBE cells	miR-218↓	IL-1β, IL-6, TNF-α↑	Song et al. (2020)
Beas-2B/HBE cells	miR-582-5p↓	HIF-1α↑	Jiang et al. (2021)

Beas-2B cells, human bronchial epithelial cell line; HAEC, human aortic endothelial cells; A549 cells, human alveolar basal epithelial cell line; 16HBE cells, human bronchial epithelial cell line; COPD, chronic obstructive pulmonary disease.

#### 2.2.1 Th17

In the presence of pro-inflammatory cytokines IL-6, IL-1, TGF-β, and IL-23, IL-21, and CD4^+^ T cells induce the expression of the RORγt gene through STAT3 and then differentiate into Th17 cells (Zhang et al., 2021). The experiment confirmed that cytokines, such as IL-23 are necessary to produce Th17. Cytokine deficiency can induce autoimmunity disorders (Cua et al., 2003). In addition, after activation of the PI3K/Akt signaling pathway, mTOR stimulates glycolysis supported by HIF-1, which also essentially drives Th17 type expression (Cong et al., 2020). Th17 cells can express a variety of pro-inflammatory cytokines, such as IL-17A, IL-17F, IL-22, and GM-CSF (Yasuda et al., 2019). It can be defined that Th17 is the hinge of autoimmune diseases (Murphy et al., 2003). Th17 immune pathway can recruit other inflammatory cells, thereby causing direct damage to the tissue.

Several studies have confirmed the vital role of Th17 in the pathogenesis of PMN. Huimin Li et al. observed that IL-17A was significantly upregulated in MN patients. Simultaneously MDSCs, part of peripheral blood mononuclear cells (PBMC), participated in disease progression through the Th17 immune pathway and inhibited Th2 polarization under the mediation of IL-6 and IL-10 (Li H. et al., 2020); Marion Cremoni et al. observed a significant increase in the expression of IL-17A in MN through *in vitro* stimulation and verified the activation of Th17, Th2, and Th1 pathway in MN after vivo stimulation, which manifested as variety expression of related cytokines (Cremoni et al., 2020). And Th17 cells are more flexible and sensitive to changes in the immune microenvironment than other effector T cell subsets (Bluestone et al., 2009). The tissue damage phenotype caused by Th17 cells is not static. But the timing of Th17 responses playing a significant role in PMN remains to be excavated, which may be more involved during relapse and progression. Roza Motavalli et al. (2021) examined the ratio of Th17/Treg in PBMCs of 30 newly diagnosed and unsuppressed PMN patients and 30 healthy people. They did not find significant changes in Th17 numbers in newly diagnosed MN patients but observed a differential reduction in Treg, resulting in an increased Th17/Treg ratio. PM2.5 particles can stimulate the production of IL-1a, which is involved in the production of inducible bronchial-associated lymphoid tissue (iBALTs). This potency is sustained (Kuroda et al., 2016), which can trigger the transition of T-cell immunity to the Th17 immune pathway. Rheumatoid arthritis, an autoimmune disease mediated by genetics and environment, is also closely related to this process (Sigaux et al., 2019). The entry of PM2.5 into the lung through the airway can induce the upregulation of IL-17A and activate the immune-inflammatory pathway to participate in the induction of lung injury (Cong et al., 2020). The increase of IL-17A is macrophage-dependent. The pro-inflammatory effects of T-cytokines under the action of macrophages are persistent (Ma et al., 2017).

#### 2.2.2 Th2

After naive T cells are stimulated by antigens and signal transduction such as T cell receptor (TCR) *in vivo*, they differentiate into Th1, Th2, Treg, and other subsets. Th2 cells mainly produce IL-4, IL-5, IL-10, and IL-13. They can mediate humoral immunity, trigger B cell activation and promote immunoglobulin synthesis.

Since the immune complex deposited in the glomerular capillary wall of PMN is accounted for IgG4 for the main part. Therefore, it is considered that Th2 is one of the main immune pathways in PMN (Kuroki et al., 2005). An evaluation of Th subgroup mRNA expression also proved that IL-4 and IL-5 mRNA were significantly increased in MN renal biopsy specimens compared with other types of glomerular diseases (Ifuku et al., 2013). A study found that Th2 immune pathway-related factors peaked between 4 and 6 h after stimulation. Then Th2 showed a downward trend (Openshaw et al., 1995; Hirayama et al., 2002; Masutani et al., 2004), thus speculating that the polarization of Th2 may be closely related to the immune activity in the early stage of MN. The initial Th2 polarization phenomenon will increase the response of B cells to promote the production of IgG4 (Kuroki et al., 2005). So Th2 is involved in the mechanism of MN immunoglobulin generation.

#### 2.2.3 Treg

Different from immune pathways such as Th17, Th2, and Th1, Treg cells differentiated from CD4^+^ naive T cells are crucial in suppressing autoimmune responses (Strom and Koulmanda, 2009). TGF-β, IL-2, and Foxp3 are cytokines necessary for differentiation into Treg. Treg cells are rapidly recruited to the injured site when inflammation occurs, secreting TGF-β and IL-10 to inhibit immune overactivation.

Treg’s percentage decreased significantly in PMN patients compared to the healthy control group. Also, Treg increased significantly 8 days after rituximab stimulation. This phenomenon means the clinical response of Treg to the drug is sensitive (Rosenzwajg et al., 2017). In addition to PMN, Treg and IL-10 also serve as critical therapeutic factors in systemic lupus erythematosus (SLE). And the severity of SLE can be reduced by increasing the number of Treg cells and the production of IL-10 (Lee et al., 2019). The PAH components in PM2.5 can damage the Foxp3 locus necessary for Treg cell differentiation by binding to cell surface AhR (Sun et al., 2020). Another study also observed a decrease in Treg cell differentiation under exposure to PM2.5 (Gu et al., 2017).

#### 2.2.4 Th17/Treg immune balance

Th17 and Treg cells are differentiated from naive CD4^+^ T cells under the influence of a microenvironment composed of shared and different cytokines. They are a novel and classic paradigm for understanding autoimmune regulation. Following Th1 and Th2, Th17 and Treg cell subsets are more representative and flexible for understanding the immune status, proving that effector T cells have a more refined division of labor. Th17 and Treg are interrelated. A variety of cellular microenvironmental factors are the most critical factors in regulating the metabolic reprogramming of CD4+T cells. For example, Foxp3, which is necessary for Treg differentiation and development, interacts with RORγt to inhibit Treg binding to DNA, thereby changing Th17 differentiation into Treg (Sun et al., 2017); TGF-β, as an initial differentiation signal, which is involved in two kinds of differentiation of immune cell lines (Zhang et al., 2021), induces iTreg cells to convert to Th17 cells under the conditions of IL-6 (Bettelli et al., 2006).

Microarray data showed that the expression of IL-17 signaling pathway mRNA in lung tissue increased under the action of PM2.5. The expression of IL-17 in lung tissue was significantly increased after PM2.5 exposure (Jeong et al., 2019). Lin et al. (2020) showed that PM2.5 intratracheal perfusion could lead to an increase in the number of lymphocytes in the lungs and a decrease in the number of TGF-β cytokines; metabolic responses and costimulatory signals are also involved in affecting this balance. Such as blocking glucose glycolysis can promote the generation of Treg cells and inhibit the development of Th17 cells (Shi et al., 2011). Dynamic changes in Th17/Treg immune balance can reflect changes in the local immune microenvironment and represent an epigenetically unstable variable whole (Strom and Koulmanda, 2009) to induce the occurrence and aggravation of PMN. Studies have confirmed the phenomenon of increased Th17 cells and decreased Treg cells in MN, showing the existence of a Th17/Treg immune imbalance, and this balance is restored after immunotherapy (Ma et al., 2021). However, the mechanism underlying PM2.5 disrupts this balance in PMN remains unclear.

There are many speculations about the key pathogenic mechanism of air fine particle pollutant PM2.5. First, they enter the respiratory system, penetrate deep into the alveoli, and diffuse to other organs through the blood-air barrier (Falcon-Rodriguez et al., 2016), resulting in the local immune-inflammatory response. They can induct pro-inflammatory cytokines release or oxidative stress in the respiratory system. These cytokines directly enter the blood circulation, thus activating NF-κB, STAT1 pathways, et al. (Falcon-Rodriguez et al., 2016), triggering a systemic inflammatory cascade (Krauskopf et al., 2018). After PM2.5 suspension was instilled in mice trachea, the increase of Th17 immune pathway polarizing factor IL-6 was observed in peripheral blood circulation. And the increase in IL-6 was PM2.5 dose-dependent (Hou et al., 2018). The cellular and molecular mechanisms of MN pathogenicity after PM2.5 enter the blood circulation have not been elucidated. There are studies evaluating the effects of PM2.5 incubation on podocytes and found that podocyte cell homeostasis and immune-related molecules were significantly affected (Wan et al., 2020); Air pollutants use the lung as the starting point of autoimmunity and can regulate Th17/Treg; Studies found that prolonged exposure to fine particulate pollutants can lead to inflammation and endothelial function-related markers in the blood intercellular adhesion molecule-1 (ICAM-1) and vascular cell adhesion molecule-1 (VCAM-1) elevate (Rui et al., 2016), which also explains the increase in blood coagulation indexes in patients with PMN. Air pollution fine particles not only affect the local but also trigger systemic effects; PM2.5 treatment of CD4^+^ naïve T cells in healthy human PBMC, similar inflammatory stimulation was observed, which can interfere with Th17/Treg balance through HIF-1α, glutamate oxaloacetate transaminase 1, as described before. Taking the complex transcriptional response of HIF-1α under hypoxia as an example, miR-210 is a hotspot closely related to it, also mediating macrophage apoptosis (Karshovska et al., 2020), Th17 differentiation (Wu et al., 2018), NF-κB pathway (Jia et al., 2020), and other effects.

**FIGURE 2:**
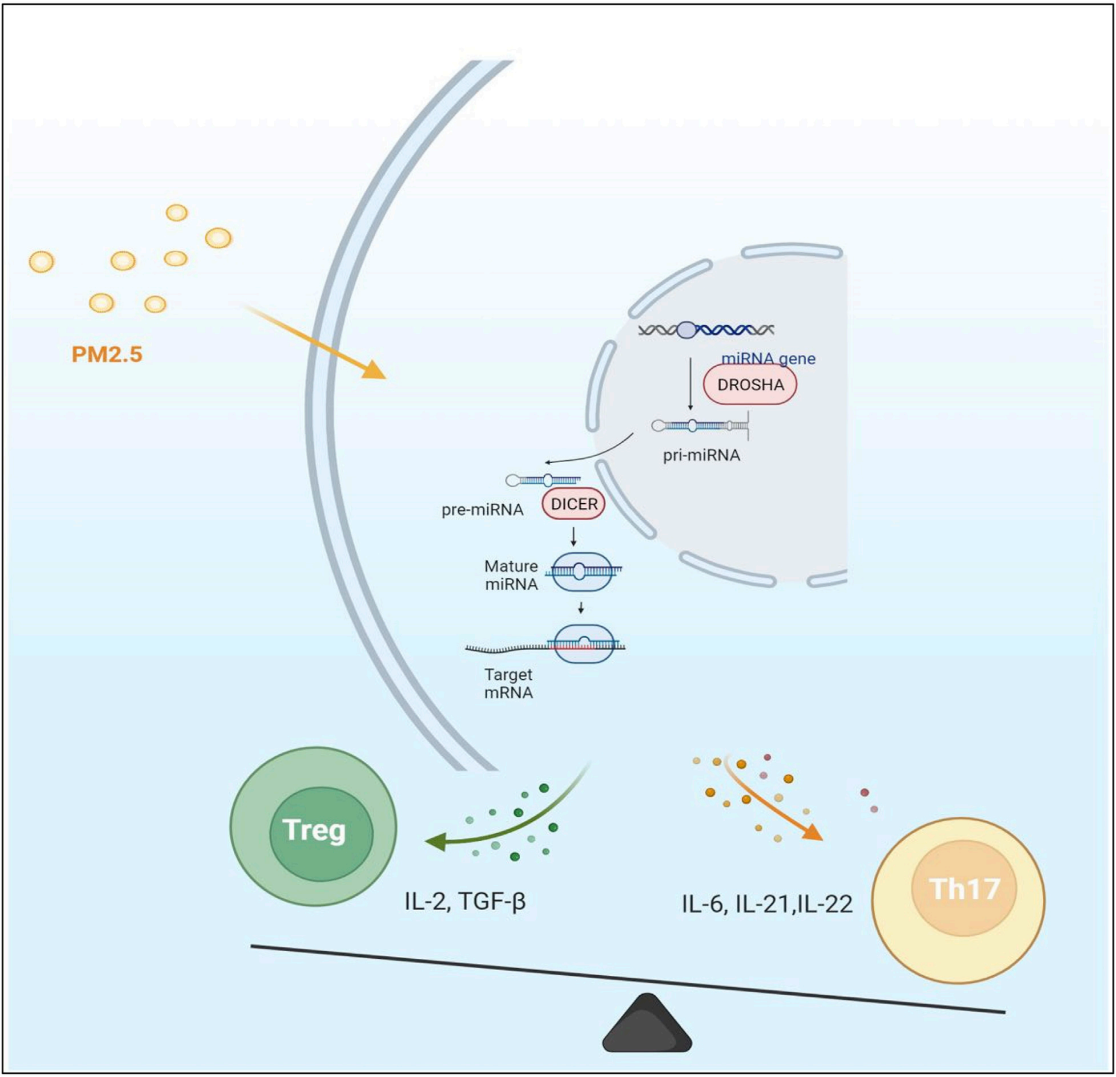
The expression of microRNA and mRNA in cells was changed under the effect of PM2.5 particles. Most of the immune factors secreted by cells are proinflammatory factors under PM2.5 stimulation. The immune balance of Th17/Treg was disturbed. The Th17 immune pathway is polarized. However, it is not clear which step of microRNA production is significantly affected by PM2.5.

As a stimulatory molecule from the external environment, PM2.5 has been confirmed to be associated with a high incidence of PMN. In terms of immunity, it has been confirmed that it can regulate microRNA and cause Th17/Treg immune imbalance in multi-system diseases. To our knowledge, there is currently no direct evidence for this pathway in PMN. There is also no data on miRNAs changes in PMN models under the effect of PM2.5. Taking miR-194 as an example, *in vitro* cell experiments confirmed the impact of PM2.5 on the downregulation of miR-194-3p (Zhou et al., 2018). qPCR analysis of renal biopsy samples from CKD patients with different disease types revealed that miR-194 was significantly downregulated in the advanced stage. MN patients were also included in the discovery cohort of this study (Rudnicki et al., 2016). However, this data on miRNAs expression in MN patients need further analysis. These two studies were tested in different tissues and did not prove our conjecture. MicroRNA is a kind of communication medium between cells and tissues. And its expression at specific sites may also provide signals. As an essential part of the epigenetic mechanism, miRNAs are also susceptible to the stimulation of PM2.5, affecting critical components of immune pathways by binding to target mRNAs (Bartel, 2004) (Figure 2). The inspiration of foreign molecules creates a stressful environment, and many baroreceptors, such as p53, direct regulatory effects on miRNAs (Krauskopf et al., 2017). In addition, other studies have found that PM2.5 can directly penetrate the placenta. Parental exposure to PM2.5 directly affects the parental Th17 and Treg immune pathways and may change the immune microenvironment in offspring. The expression of IL-17A, IL-6, and IL-10 in offspring significantly increases with or without exposure to PM2.5, suggesting that the Th17/Treg balance was indirectly affected, which is called genetic susceptibility (Zhang et al., 2019).

## 3 Composition and mechanism of particulate matter 2.5

PM2.5 is a kind of fine particle pollutant with an aerodynamic diameter ≤2.5 μm, which is the main component of smog, mainly released from mineral combustion, automobile exhaust emissions, and construction industry processes. Its large surface area can absorb various toxic substances (Perrone et al., 2013). Taking Hangzhou, China, as an example, PM2.5 in this city is primarily composed of metals and organic ingredients like PAH. The concentration of PM2.5 increases in Hangzhou is related to the increase in construction sites, traffic emissions, and road asphalt particles (Fu et al., 2020); In rural and suburban areas, however, the harmfulness of PM2.5 pollution is sometimes overlooked. Taking the rural and remote mountainous areas of Milan and Italy as examples, in this study, the metal components of PM2.5 are mainly produced from aerosols and natural dust sources, also including organic elements such as PAH. But the biological effect of PM2.5 may be higher than that of PM1. It is easier for PM2.5 to cause DNA damage (Perrone et al., 2013); In a specific working environment, taking the subway as an example, the components of PM2.5 are mainly iron, copper, manganese, and other metals. The concentration of PM2.5 particles is thicker than the surface, affected by season and the burning of minerals. The concentration will increase in winter when the need for warmth is higher (Chang et al., 2021). PM2.5 particles have a large surface area. They enter the lung through the respiratory system and act on other human body organs. The results of a prospective study spanning 2005 to 2018 and covering 121 counties in Hunan, China, showed a strong positive correlation between high-level PM2.5 exposure and chronic kidney disease (Duan et al., 2022). Animal studies have shown that the kidney is a toxicological target of particulate pollutants inhaled from the lungs (Nemmar et al., 2010). PM2.5 exposure is associated with early kidney injury. This effect is not only reflected in the damage to the endocrine function of the kidney (Aztatzi-Aguilar et al., 2015) but also in the increase of the renal damage markers β-2-microglobulin and cystatin-C (Aztatzi-Aguilar et al., 2016). The toxic effects of metal and PAH elements are closely related to oxidative stress (Greenwell et al., 2002), Th17 immune pathway activation (Zhang et al., 2018b), and gene transcription changes (Mehta et al., 2008). Previous studies on PM2.5 hazards used filters to collect PM2.5 particles. They can analyze the components carried on the surface of the particles. It is achieved by conducting *in vivo* or *in vitro* experiments such as tracheal instillation of PM2.5 suspensions or real-world ventilation. As a particulate matter that can enter the alveoli, PM2.5’s significant impact on human immunity and epigenetics is not only related to the toxicity of its surface adsorption components. Akiko Honda et al. (2021) avoided the contamination of PM2.5 ingredients by extracting materials through cyclone separation technology, thus analyzing the effect of PM2.5 as a powder itself on immune cells and factors. Compared with the organic components of PM2.5, it has a more noticeable impact on reducing cell viability and promoting the release of IL-6.

### 3.1 Particulate matter 2.5 and the onset of primary membranous nephropathy

Due to its small diameter, unlike the large particles cleared away by mucociliary, PM2.5 can accumulate in inhaled alveoli, be phagocytosed by alveolar macrophages, be absorbed by pulmonary vascular endothelial cells, or along with the lung’s air exchange entering the circulatory system (Xing et al., 2016). Air pollution is closely related to the pathogenesis of multi-system diseases such as respiratory and cardiovascular systems. This capability has a significant impact in many developing countries. The impact of PM2.5 on the incidence and progression of many diseases has been confirmed. It is known that both PM2.5 pathogenicity and glomerular diseases have regional and ethnic differences. However, the role of PM2.5 from different regional sources is different. This phenomenon may be related to varying sources of PM2.5 (Table 2). Related articles have confirmed that higher concentrations of PM2.5 in different regions will increase the risk of MN. A study covering a database of 43.7 million hospitalized patients in China confirmed the influence of environmental and geographical factors on the incidence of PMN. This study takes the Yangtze River as the geographical boundary. The incidence of PMN in the north of the Yangtze River was positively correlated with PM2.5 exposure. While the incidence in the south of the Yangtze River is positively correlated with the proportion of the Zhuang population, but not PM2.5 exposure (Li et al., 2018b). A study covering 938 hospitals in 282 cities in China showed that in areas where PM2.5 concentrations >0.70 mg/m3, every 10 mg/m3 increase in PM2.5 concentration increases the risk of MN by 14%. In addition, during the past 10 years, unlike other kinds of glomerular diseases that remained stable disease incidence, the incidence of MN has increased by 13% every year (Xu et al., 2016). It is possibly related to the increased air pollution condition in recent years.

**TABLE 2:** The disturbed miRNAs and their target mRNA after exposure with PM2.5 in different areas.

Source of PM2.5	microRNA change	Target mRNA	Changed factor	References
SRM 1648a	miR138-1-3p↓	—	IL-6, IL-8, NF-κB↑	Jin et al. (2021)
Beijing, China	miR-21-5p↑	SOX7	VE-cadherin↓	Shiwen Wang et al. (2020)
SRM 1648a	miR-582-5p↓	HIF-1α	HIF-1α↑	Jiang et al. (2021)
Boston area, United States	8 miRs↓	—	HMGB1/RAGE↑	Fossati et al. (2014)
Wuhan, China	miR-182/185↓	SLC30A1, SERPINB2, AKR1C1	SLC30A1, SERPINB2, AKR1C1↑	Liu et al. (2015b)
Hong Kong SAR, China	miR-125a-3p↓	TCTP	TCTP↑	Liu et al. (2020)
Beijing, China	miR-326-3p↑	IκBα	TNF-α, NF-κB, VCAM1↑	Gao et al. (2022)
Beijing, China	6 miRs	—	E-cadherin↓ Vimentin↑	Yunxia Wang et al. (2021)
Purchased from United States	miR-16-1-3p↓	Twist1	Twist1↑	Zhang and Li, (2019)
Jiangsu, China	miR-29b-3p↑	PI3K(P85a)	P53↑PEAMIR↓ PI3K(P85a)/ATK/GSK3b↓	Pei et al. (2020)
Shenyang, China	miR-32↑	Smad1	Smad1↓	Yang et al. (2017)
Beijing, China	miR-139-5p↓	Notch1	Notch1↑	Yunxia Wang et al. (2020)
Beijing, China	3 miRs↑	—	IL-4↓, IFN-γ↑	Hou et al. (2018)
Beijing, China	miR-149-5p↓	TAB2	MAPK, NF-κB↑	Qiuyue Li et al. (2021)
Zhengzhou, China	miR-155↑	FOXO3a	FOXO3a↓	Jiansheng Li et al. (2021)
Shijiazhuang, China	miR-183/96/182↑	FOXO1	NACHT, LRR, NALP3↑	Lixiao Zhou et al. (2019)
Taiyuan, China	miR-206↑	SOD1	CAT/GSH/GSH-Px/T-SO↓ ROS↑	Lei Wang et al. (2019)
Changchun, China	miR-331↓	IKK-β	NF-κB↑	Song et al. (2017)
Shanghai, China	miR-338-3p↓	UBE2Q1	UBE2Q1/AKT/mTOR↑	Jin-Chao Wang et al. (2021)
Purchased from United States	miR-486↓	PTEN, FOXO1	PTEN, FOXO1↑	Jin Li et al. (2018)
Taiyuan, China	miR-574-5p↓	BACE1	BACE1↑	Ku et al. (2017)

SRM, 1648a: The standard reference material (SRM) is atmospheric particulate matter collected in an urban area and acts as a control material used in the inorganic analysis of atmospheric particulate matter. A unit of SRM, 1648a consists of a bottle containing 2 g of atmospheric particulate matter.

Current studies believe that genetic risk factors determine most glomerulonephritis; MN is no exception which has been shown to have susceptibility to HLA-DQ, HLA-DR, and PLA2R1 genes. More importantly, anti-PLA2R1 antibodies were detectable in 73% of patients with high-risk genotypes of PLA2R1 and HLA-DQA1, which differed from the low-risk population (Stanescu et al., 2011; Gupta et al., 2018). The effect of PM2.5 on PMN incidence may also be premised on genetic susceptibility through stimulation of immunity or exposure to self-antigens. Risk amino acids encoded by risk alleles DRB1*1501 and DRB1*0301 of PMN have been reported to promote the role of T cell epitopes in the presentation of PLA2R1 antigen by MHC class II molecules in the Chinese Han population (Cui et al., 2017). Currently, no evidence shows whether PM2.5 is more related to PLA2R1-positive MN. Perhaps because of its large proportion, unique biological characteristics, and different conformation, most studies have focused on PLA2R1. The relationship between other specific antigens of MN and environmental pollution needs more studies to verify. We hypothesized that the effect of PM2.5 on the increased incidence of MN may not be limited to specific autoantigens but may be related to the population with risk genes. And this susceptibility may be achieved through epigenetic pathways.

Although epidemiological evidence suggests that PM2.5 contributes to the increased incidence of PMN, no convincing data has confirmed the molecular mechanism of this process. Studies observe that PM2.5 can deposit in the kidney through macrophage endocytosis or adsorption of proteins, resulting in damage to the glomerulus (Saint-Georges et al., 2008). But the onset and progression of PMN are inseparable from exposure to self-antigens. Therefore, the speculation that air pollution can lead to self-antigen exposure is more valuable. The resulting anti-PLA2R1 antibodies first bind with high affinity to the antigen in the kidney. Circulating anti-PLA2R1 antibody concentrations are not detectable until the kidney binding sites are saturated (van de Logt et al., 2015). In addition to the relationship between its antibody concentration and disease progression (Beck et al., 2011), changes in the PLA2R1 epitope are also closely related to disease progression and patient subgroups (Fresquet et al., 2015). There are several hypotheses about how PM2.5 causes PLA2R1 exposure. PM2.5 can trigger vascular endothelial cell inflammation through toll-like receptors (TLRs) or by stimulating macrophages to make them a potential phospholipase A2 (sPLA2) target. These effects may contribute to the exposure of PLA2R1 that circulates continuously between the plasma membrane and the endosome (East and Isacke, 2002; Ronco and Debiec, 2012); Studies by Paul D. Boyce et al. (2006) demonstrate that inhalable metal particles alkalize the airways and increase pH. Compared with an acidic environment, PLA2R1 will appear conformational extension in the alkaline environment (Dong et al., 2017) (Figure 1A); PM2.5 causes oxidative stress, and the oxidative cellular microenvironment may cause PLA2R1 to retain disulfide bonds (Liu et al., 2019). In addition, the PAH components in PM2.5 can cause polarization of the Th17 immune pathway through AhR (Sun et al., 2020), reducing Tregs (Figure 1C). The balance of immune tolerance is disrupted. Small amounts of anti-PLA2R1 antibodies are inherently present in the circulation. Under the premise that the tolerance balance is broken, PM2.5 particles disturb the balance of PLA2R1 and antibodies, which may make the kidneys more severely hit. PM2.5 may also promote PLA2R1 exposure by activating AhR on podocytes (Li et al., 2018b). And AhR is believed to be involved in various responses such as immune inflammation by altering the epigenetic mechanism of miRNAs (Disner et al., 2021). However, there are few studies on this process in PMN.

PM2.5 particles first act on the human body through the lung tissue. And the PLA2R1 formed in the lung stimulates the immune system to generate anti-PLA2R1 antibodies, which then act on the kidneys through circulation. It is unclear whether organs other than the lung express PMN autoantigens represented by PLA2R1. However, the roles of PM2.5 particles are limited. No studies have confirmed that PM2.5 is necessary for the pathogenesis of PMN. Whether large inhalation or chronic exposure to PM2.5 particles, the major impact is the occurrence of respiratory diseases such as asthma (Yu et al., 2022). Therefore, the contribution of PM2.5 to the incidence of PMN may be achieved based on the production of nephrogenic antibodies.

### 3.2 Intervention of particulate matter 2.5 on immune balance and miRNAs expression

PM2.5 particles are inhaled into the lung from the airway. Part of them can be directly translocated to the alveolar extravascular matrix and vascular endothelial cells. Thereby PM2.5 particles enter the systemic circulation and cause miRNAs changes related to various inflammatory pathways. Bioinformatics analysis showed that changes in respiratory system function caused by short-term exposure to PM2.5 are mainly concentrated in immune pathways (Lin et al., 2022). After passing through the respiratory system, PM2.5 can enter the blood circulation. Studies confirmed that PM2.5 particles are related to the increased expression of Th17 immune pathway-related factor IL-17A in MN. Marion Cremoni et al. found that PM2.5 can be used as a threshold for distinguishing IL-17A positivity at the concentration was 58 pg/ml (Cremoni et al., 2020). In the case of the podocyte *in situ* antigen exposure in PMN patients, changes in immune activity represented by Th17/Treg immune balance can form autoantibodies against situ antigen. The role of PM2.5 in influencing Th cell differentiation in multiple ways has been confirmed by many studies, such as acting on Th17/Treg balance through AhR (Sun et al., 2020), NF-κB (Wan et al., 2020), Notch (Gu et al., 2017), et al. Oxidative stress is one of the important responses of PM2.5 intervention. Elevation of NF-κB under PM2.5 stimulation activates pro-inflammatory responses under oxidative stress (Longhin et al., 2016). The abnormal expressions of miRNAs caused by PM2.5 mainly focus on the changes in NF-κB. What is the significance of miRNAs in the differentiation of Th cells? How do miRNAs work?

Epigenetic regulation regulates gene expression at the transcriptional and translational levels without altering the nucleotide sequence (Balasubramanian et al., 2020). The occurrence and progression of MN are closely related to the environment, genetics, and immunity. These different factors all contain the abnormal expression of miRNAs stimulated by external environmental toxins. As a critical mediator in regulating gene expression at the transcriptional translational level, miRNAs function as an essential aspect of epigenetics (Bartel, 2004). Several studies have confirmed the connection between miRNAs and external toxins, such as ethanol (Pasqualotto et al., 2021) and cigarette smoke (Donate et al., 2021), et al.

The differentiation of Th cells, which have plasticity, is affected by antigens and co-stimulatory factors in the cellular microenvironment. Different types of Th cell differentiation are often accompanied by relatively specific miRNAs molecular patterns. After the stimulation of TCR, downstream signaling levels are activated in multiple ways. In this process, miRNAs act as regulatory targets and upstream regulatory factors to control the differentiation of effector T cells (Zhou et al., 2007). The regulation of critical factors’ mRNA that governs signal threshold plays a considerable role in the differentiation and development of T cells (Baumjohann and Ansel, 2013). The previous study about the changes of miRNAs in PMN has found that downregulation trend in most cases. Mechanistically, it may be explained that T cells are stimulated to induce AGO2 ubiquitination and its proteasome-dependent reversal, shortening the half-life and leading to an overall decrease in the abundance of miRNAs (Bronevetsky et al., 2013). That is why the expression of corresponding stress factors and mRNA will increase. In the state of pathological stress, the role of microRNA can be said to be small because it has a considerable number of targets and has multiple regulatory effects on the downstream multi-level cascade after TCR activation (Baumjohann and Ansel, 2013). The diversity of its roles and the coincidence with the target also makes its function impossible to detect by conventional experimental means, obscuring its important regulatory significance.

## 4 MicroRNA: Biological function and action formation

### 4.1 MicroRNA production and biological function

MiRNAs are a class of small endogenous single-stranded non-coding RNAs about 22 nucleotides in length, repressors of post-transcriptional gene regulation. MiRNAs first experience gene encoding in the nucleus and form the classical hairpin precursor. However, they are not directly involved in encoding proteins since they negatively regulate gene expression through complementary pairing with the 3′UTR region of mRNA, causing mRNA translation inhibition or inducing its degradation to negatively regulate gene expression (O'Brien et al., 2018) (Figure 2). Single miRNA can target multiple mRNAs, yet multiple miRNAs also can synergistically work on one single mRNA, hinting the roles of miRNAs in life processes are diverse and complex. Considering such features, changes in multiple signaling pathways stimulated by environmental pollution can be displayed by miRNAs and act downstream. Therefore, miRNAs may represent the body’s stress response to stimulation. MiRNAs themselves are not easily degraded, primarily located inside cells. Another part called circulating miRNAs is located in the extracellular environment, which can circulate in body fluids related to RNA-binding proteins or in exosome-like lipid vesicles. As regulators of post-transcriptional processes, miRNAs play crucial roles in various cellular processes of development, proliferation, differentiation, and apoptosis, as well as stress responses (Iorio et al., 2010). They are thought to be involved in epigenetic mechanisms as an important player in gene expression regulation. Studies have found that the progression of MN and immune disorders are closely related to the functional changes of some miRNAs. Environmental and health research results indicate that epigenetics may mediate environmental factors’ effects at the gene regulation level. Liu et al. (2015a) prove that epigenetic differential markers caused by environmental pollution exist in genes and are related to immune activation. MiRNAs are epigenetically sensitive response molecules to external pollutant stimuli (Balasubramanian et al., 2020). They can link air pollution with induced immune activity. MiRNAs play a vital role in the inflammatory activity of macrophages involved in the phagocytosis of PM2.5 (Hamidzadeh et al., 2017). The abnormal expression of miRNAs caused by exogenous pollutants can be reflected in circulating miRNAs (Balasubramanian et al., 2020).

### 4.2 MicroRNAs as membranous nephropathy diagnostic markers

Diagnosis of MN and other types of kidney disease largely depends on the typical injury pattern in biopsy tissue shown by microscopy. Renal biopsy, although important in confirming the type of disease, can still cause a certain degree of kidney damage as an invasive test. There is an urgent need for a non-invasive method to judge and predict disease progression. MiRNAs have made some progress in this field. A group of highly kidney-specific miRNAs cluster molecules has been found by K-means clustering, which are miR-192, miR-194, miR-204, miR-215, and miR-216 (Sun et al., 2004). Possibly because of the close distance, they regulate gene expression as a common transcriptional unit. MiR135a-5p, 146b5p, 150-5p, and 155-5p can be used as risk markers for chronic kidney disease (CKD) nephropathy progression by reflecting the degree of renal fibrosis or established renal failure (Pawluczyk et al., 2021). Unlike healthy people or other diseases, PMN patients have many differentially expressed miRNAs. Research results in recent years have shown that differentially expressed miRNAs have important roles in mediating inflammation, immune response and provide diagnostic value. Many studies have found that multiple miRNAs expression and function are altered in MN patients. In a 2018 study, ten microRNAs were found differentially expressed in biopsies from patients with MN compared with healthy controls. They target IL-6 and MYC, which are related to immune response; Among them, miR-204 can not be ignored in the immune process of MN for suppressing T cell proliferation (Barbagallo et al., 2019). MiR-27b-3p and miR-1228-3p were significantly upregulated in renal biopsy tissue of diabetic membranous nephropathy patients instead of diabetic nephropathy patients (Conserva et al., 2019). However, in this study, there was no direct analysis of the difference in expression of these two miRNAs between diabetic membranous nephropathy patients and healthy subjects. At present, no more studies on miR-27b-3p in MN have been retrieved. Based on the miRNAs microarray dataset GSE51674 downloaded from GEO, Yawei Hou et al. (2021a) extracted the MN and healthy control group information and combined it with the mRNA microarray dataset GSE108109 for target gene prediction. Finally, they screened out ten differentially expressed miRNAs involved in processes such as podocyte autophagy and renal fibrosis in MN by targeting mRNA regulatory networks. There is also a significant downregulation of miR-217 in MN compared with healthy people, which targets the expression of tumor necrosis factor superfamily member 11 (TNFSF11). MiR-217 has sensitivity and specificity for distinguishing MN patients from healthy people (Li et al., 2017). Based on the detection value of serum anti-PLA2R1 antibody for disease, the serological detection method, as a non-invasive method, can play an important role in predicting the occurrence and prognosis of PMN (De Vriese et al., 2017). Different from the invasive detection method of kidney biopsy tissue, the detection of diagnostically valuable factors in serum is less invasive to the human body. miRNAs can exist stably in various body fluids. The studies of miRNAs sequencing in serum as a biomarker have also found a lot. InO.Sun et al. (2022) detected the expression of miRNAs in serum extracellular vesicles by RNA sequencing method. The identification results showed that the expression levels of miR-1229-3p, miR-340-3p, and miR-99b-5p in PMN differed from those in idiopathic nephrotic syndrome and healthy people. Differentially expressed miRNAs in extracellular vesicles can reflect the clinical treatment response of patients. Different humoral-derived miRNAs are differentially expressed in PMN. MicroRNA database analysis revealed differentially expressed miR-195-5p and miR-328-5p in MN and healthy people target PPM1A and BRSK1, respectively. These two target genes are associated with MAPK pathway activation. In addition, there is also miR-192-3p involved in the p53 signaling pathway by targeting RAB1A (Zhou et al., 2019a). MicroRNAs exist stably in peripheral blood. These miRNAs released from cells into the circulation may represent an overall profile of differential expression under stress. PBMCs from 30 adult patients with MN were collected from the nephrology department of a hospital in Iran and compared with healthy controls. MiRNAs-sequencing of collected PBMC samples by real-time PCR revealed a significant increase in miR-30c and miR-186 (Hejazian et al., 2020). MiRNAs in PBMC can not only distinguish MN from healthy people but can also distinguish MN from other glomerular lesions. For example, miR-106a-5p and miR-30a-5p can distinguish mesangial proliferative glomerulonephritis from MN (Wang et al., 2020a). By analyzing morning urine samples, Jinshi Zhang et al. (2020) found 28 specifically expressed miRNAs in the urinary exosomes of PMN patients. The target genes of these miRNAs are widely involved in pathway responses such as podocyte autophagy and macrophage inflammation. 2022, in Datong, Shanxi, Songjia Guo et al. (2022) run high-throughput miRNAs sequencing of urinary exosomes. Results showed that miR-30b-5p and miR-9-5p were significantly downregulated in PMN patients compared with healthy people. Moreover, in this study, miR-30b-5p in urinary exosomes was significantly correlated with the clinical indicator anti-PLA2R1 in serum. By analyzing the urinary sediment microRNA database GSE64306 in previous studies, Guangyu Zhou et al. (2021) compared the data of MN and healthy people. Data analysis showed that five miRNAs, miR-145-5p, miR-148a-3p, miR-148b-3p, miR-3605-5p, and miR-497-5p, could participate in the immunity and metabolism of MN through their target mRNAs Regulatory Network.

### 4.3 MicroRNAs and immune system cells

MiRNAs are essential kinds of immune cell regulators. As a kind of factor that has a clear effect on the stability of Th cells (Zhou et al., 2008), miRNAs have an important impact on the development and function of the immune system. They participate in the disease process by targeting a variety of mRNAs. PM2.5 first invades the respiratory system. In some lung diseases, it has been confirmed that miRNAs affect the balance of immune pathways such as Th1 and Th2, which occupy the leading position and further damage the immune system (Hou et al., 2018). MiRNAs can participate in the effect of PM2.5 by targeting immune-inflammatory-related factors, which has been demonstrated in multiple systems. PM2.5 inhaled into the lungs has been confirmed to play oxidative stress on lung tissue through miRNAs targeting function, activating NF-κB, NLRP3 (Zhou et al., 2020), and M1 polarization (Zhong et al., 2019). While causing lung damage, inflammatory factors secreted by these processes can enter the bloodstream and cause changes in miRNAs. Gene-environment interactions in PMN are important regulators of immune imbalance. The expression of genes involved in the inflammatory signaling pathway response is significantly increased in active PMN (Xu et al., 2021), which is considered to be related to the post-transcriptional negative regulation of miRNAs. Studies have shown that miR-16 can regulate various immune-related factors, such as IL-6, TNF-α, IL-4, IL-8, et al. These factors can affect the balance of Th17 and Treg. MiR-16 is involved in the immune process of SLE. It is highly expressed in PBMC and can be used as a recurrence marker of SLE (Yan et al., 2019). As mentioned above, miR-27b-3p was significantly upregulated in diabetic membranous nephropathy patients compared with patients with diabetic nephropathy. However, in a recent study on the function of miR-27b-3p, it was found that miR-27b-3p was significantly downregulated in chicken peripheral blood lymphocytes treated with ammonia, a component of PM2.5 aerosol, *in vitro*. Treg immune pathway-related protein gene expression was significantly decreased. Th2 and Th17 immune pathway-related protein expression were significantly increased (Zhang et al., 2022). In the treatment aspect of MN, studies have reported that tacrolimus, a calcineurin inhibitor, has an immunosuppressive effect on the disease. But its efficacy varies significantly among individuals, which may be related to the regulatory effect of miR-582-5p. MiR-582-5p is altered to affect the expression of PPP3R1 (Zhu et al., 2018).

### 4.4 MicroRNAs’ damage to podocytes and kidneys

Podocytes are important presenting cells for *in situ* antigens as a relatively fully differentiated cell line. MN is marked by podocyte apoptosis. Although miRNAs are not much involved in developing human cell lineages and tissues, they play a central regulatory role in fully developed cells under stress and injury conditions (Mendell and Olson, 2012). MiRNAs can affect podocytes through multiple pathways. As previously described, miR-217 is downregulated in MN. MiR-217 can target TNFSF11 to participate in podocyte apoptosis. Upregulation of this miRNA *in vitro* attenuates damage to podocytes. Ling-wei Jin et al. (2019) demonstrated that the effect of miR-217 on podocyte apoptosis in MN could also be achieved through TLR4. Sha et al. (2015) revealed that miR-186 was significantly downregulated in MN and promoted podocyte apoptosis. This phenomenon is accompanied by an increase in the receptor family TLR4, which is crucial for immune system activation. MiR-124 induces podocyte adhesion damage by targeting integrin α3 and β1 under stress (Li et al., 2013); Downregulation of miR-500a-5p in MN is associated with promoting podocyte apoptosis. This effect is achieved through the Circ_0000524/miR-500a-5p/CXCL16 signaling pathway (Sun et al., 2021). MiRNAs have also been proved by many studies that their normal expression plays an important role in mature podocyte homeostasis. Such as podocyte-specific deletion of Dicer or Drosha, reduced miRNAs expression can cause podocyte and glomerular dysfunction (Zhou et al., 2008). There are also miR-30 that protect podocytes by inhibiting the toxic Notch1 or p53 pathways (Wu et al., 2014). Kidney biopsies from both MN and focal stage glomerulosclerosis (FSGS) patients showed increased expression of miR-378a-3p, distinguishable from diabetic nephropathy and IgA glomerulonephritis. MiR-378a-3p can increase in podocytes under TGF-β stress and target glomerular nephronectin (NPNT) inhibition, which is associated with podocyte reduction and proteinuria in active glomerular disease (Müller-Deile et al., 2017). Podocyte-derived miR-378a-3p and glomerular-derived miR-192-5p can also jointly upregulate NPNT (Müller-Deile et al., 2021). MiR-106a, miR-19b, and miR-17 are important regulators of pro-apoptotic gene expression. Lina Wu et al. (2021) found that these miRNAs in serum targeted inhibit phosphatase and tensin homolog deleted on chromosome ten (PTEN) expression. This process may be related to the decline of glomerular filtration function by promoting podocyte apoptosis in the early stage of MN. Peng Li et al. (2021a) searched the GSE133288 database and found 20 miRNAs that were differentially expressed in the tubulointerstitial transcriptome of MN and healthy individuals. They analyzed that the SRY-Box Transcription Factor4 (Sox4) gene was significantly upregulated in MN and was targeted by miR-204-5p. In 2014, high-throughput sequencing analyzed the expression profiles of miRNAs in PBLC of MN patients and healthy people. 286 of the 326 differentially expressed miRNAs were downregulated. This is different from other kidney diseases, where upregulated miRNAs are predominant. However, like other kidney diseases, downregulation of miR-23b, miR-24, and miR-26a also leads to rapid progression of marked glomerular and tubular damage in MN (Chen et al., 2014). MiR-150-5p positivity was observed in proliferating mesangial cells, atrophic tubules, and nodular infiltrates in MN (Pawluczyk et al., 2021). The expression of this miRNA is closely related to the development and differentiation of T and B cells (Zhou et al., 2007). In a rat model of MN, upregulation of miR-193a may affect the expression of important proteins in the cleft septum of podocytes by targeting WTI. Inhibition of miR-193a is helpful for the stability of podocyte structure and glomerular barrier function (Li et al., 2019).

### 4.5 Particulate matter 2.5 and microRNA

Based on environmental epidemiological studies, it has been demonstrated that exposure to PM2.5 has a clear association with the expression of some inflammation-related miRNAs (Bhargava et al., 2019). The toxicogenomics of microRNA is closely related to the pathogenesis and progression of the disease (Piletič and Kunej, 2016). By studying the analysis of circulating miRNAs genes in human populations exposed to PM2.5, it has been summarized by Julian Krauskopf et al. that PM2.5 can target the lung, heart, kidney, and brain. Rigorous statistical analysis has demonstrated dose and pollutant species-dependent changes in circulating miRNome after 2 h of ambient air pollution (Krauskopf et al., 2018). Multiple studies demonstrated that PM2.5 exposure could affect Th17/Treg balance by affecting microRNA expression. For example, PM2.5 exposure can downregulate miR-338-3p, miR-338-3p targets UBE2Q1 to inhibit autophagy, thus disrupt Treg/Th17 balance (Wang et al., 2021a).

PM2.5 and other ultrafine particle pollutants acting on cells *in vitro* can cause oxidative stress and lead to prolonged activation of the NF-κB pathway. NF-κB pathway consistently mediates pro-inflammatory responses that release IL-6, as the switch of epigenetic changes (Bhargava et al., 2019). And vice versa is also true. For example, PM2.5 indirectly targets and participates in FoxO and P13K/Akt signaling pathways by regulating some miRNAs (Wang et al., 2019a), which are also involved in the differentiation of Th cells. Lei Song et al. (2016) demonstrated the pro-inflammatory effect of the miR-let-7 family under PM2.5 particle pollution induction. In bronchial cells, PM2.5 exposure significantly suppressed the expression level of miR-let-7a. Let-7a can inhibit arginase 2 (ARG2) to reduce oxidative stress caused by air pollution. Additionally, Iliopoulos et al. (2009) described a positive feedback loop of inflammatory factors involving the let-7 family. Activation of NF-κB and IL-6 inhibits the let-7 family. Therefore, the targeted inhibition of IL-6 and RAS by the Let-7 family is weakened. And RAS can further activate IL-6. Finally, let-7 is further inhibited. Moreover, it has been confirmed that the entry of PM2.5 particles into the body can activate the NF-κB pathway. Therefore, even a tiny amount of PM2.5 stimulation may trigger huge physiological effects driven by microRNA (Ebert and Sharp, 2012). Cytokines produced by these immune responses in the respiratory system can enter the circulation or reach the kidneys. The significant contribution of PM2.5 to PMN incidence highlights the promoting regulatory effect of miRNAs.

PM2.5 has a significant impact on the expression of immune factor-related miRNAs. The regulation of miRNAs can also directly change the expression of immune-inflammatory factors. They also have the effect of reducing the susceptibility to air pollutants in the opposite direction. Under the influence of PM2.5, sICAM-1 and sVCAM-1 levels were lower in rs1062923 homozygous carriers (Wilker et al., 2011). Some western or traditional Chinese medicine ingredients also reduce the effects of PM2.5, such as apigenin. Apigenin can regulate IL-17 and NF-κB to balance the Th2/Th17 immune disorder caused by PM2.5 (Pang et al., 2019). Indirect exposure to PM2.5 in a short time may not result in significant changes in microRNA levels. In contrast, direct exposure to PM2.5 can typically be observed with significant effects on cells, tissues, and related factors in renal and cardiovascular studies. Considering during a short exposure time, the irritating effect may not be noticeable. However, in the respiratory system, indirect exposure to high concentrations of PM2.5 in the environment can lead to significant changes in microRNA expression in nasal mucosa or endothelial cells. As previously mentioned, the regulation of PM2.5 significantly affects miRNAs expression. This is also consistent with significant changes in miRNAs in PMN. However, no detailed studies have confirmed the specific role of miRNAs in the PMN process. The effect of PM2.5 on morbidity and immune disorders *in vivo* is presumed to be related to PLA2R1 exposure. While the exposure of PLA2R1 is usually inhibited by miRNAs and stimulated by ROS (Sukocheva et al., 2019) (Figure 1B). Currently, there are few studies on the related miRNAs targeting PLA2R1 in PMN. And it is unclear which immune sites are targeted by the differentially expressed miRNAs in PMN. For example, whether miRNAs targeting PLA2R1 in PMN are involved in the Th17/Treg immune pathway remains obscure.

## 5 Conclusion

MN is an autoimmune disease mediated by environment, heredity, and immunity. PM2.5, an air pollutant that can be deposited at the bottom of alveoli, can trigger oxidation, release inflammation-related factors and promote the expression of pathogenic antigens *in situ* in trachea, bronchus, and alveoli. These products entering the bloodstream can alter the immune microenvironment and may even induce the production of circulating autoantibodies. PM2.5 particles entering the circulation can also cause Th immune imbalance, which acts on podocytes and destroys kidney function. However, the renal toxicity of PM2.5 may require the guidance of genetic susceptibility. But this effect may be related to racial genetic differences. The obvious correlation between PM2.5 and the incidence of multiracial PMN may be reflected by miRNAs. MicroRNA, a highly conserved molecule in the human body and an intermediary factor of environmental effects, is sensitive to stress responses caused by external poisons. The disturbances in microRNA are closely related to Th17/Treg immune imbalance. According to existing studies, PM2.5 can enter the human blood circulation through the respiratory system to stimulate the glomerulus, the placenta, and other trachea or viscera, leading to oxidative stress, autophagy, and immune response. They even have the effect of immune directional induction. Metal component on the surface of PM2.5 particles is related to oxidative stress through free radical action. At the same time, PAH may triggers T cell polarization or affect antigen expression through the AhR receptor. Different components of PM2.5 may also influence its pathogenic effect on PMN. However, the role of miRNAs in PMN needs more research and exploration.

## Data Availability

The original contributions presented in the study are included in the article/supplementary material, further inquiries can be directed to the corresponding author.
