# Corrigendum: MicroRNAs: Potential mediators between particulate matter 2.5 and Th17/Treg immune disorder in primary membranous nephropathy

**DOI:** 10.3389/fphar.2023.1166591

**Published:** 2023-02-27

**Authors:** Xiaoshan Zhou, Haoran Dai, Hanxue Jiang, Hongliang Rui, Wenbin Liu, Zhaocheng Dong, Na Zhang, Qihan Zhao, Zhendong Feng, Yuehong Hu, Fanyu Hou, Yang Zheng, Baoli Liu

**Affiliations:** ^1^ Beijing University of Chinese Medicine, Beijing, China; ^2^ Beijing Hospital of Traditional Chinese Medicine, Capital Medical University, Beijing, China; ^3^ Shunyi Branch, Beijing Hospital of Traditional Chinese Medicine, Beijing, China; ^4^ Beijing Institute of Chinese Medicine, Beijing, China; ^5^ School of Life Sciences, Beijing University of Chinese Medicine, Beijing, China; ^6^ School of Traditional Chinese Medicine, Capital Medical University, Beijing, China; ^7^ Pinggu Hospital, Beijing Hospital of Traditional Chinese Medicine, Beijing, China; ^8^ School of Traditional Chinese Medicine, Changchun University of Chinese Medicine, Changchun, China

**Keywords:** PM2.5, PLA2R1, microRNA, Th17/Treg, primary membranous nephropathy (PMN)

In the published article, there was an error in **Affiliations** [1, 2]. Instead of “^1^Beijing Hospital of Traditional Chinese Medicine, Capital Medical University, Beijing, China, ^2^School of Traditional Chinese Medicine, Beijing University of Chinese Medicine, Beijing, China,” it should be “^1^Beijing University of Chinese Medicine, Beijing, China, ^2^Beijing Hospital of Traditional Chinese Medicine, Capital Medical University, Beijing, China.”

In the published article, there was an error in the **Funding** statement. The number of the first fund was incorrect. The correct **Funding** statement appears below.

